# Depression among pregnant women with husbands abroad: Case control study in hostile region of AZAD jammu and kashmir

**DOI:** 10.1192/j.eurpsy.2021.848

**Published:** 2021-08-13

**Authors:** U. Zubair, S. Ali

**Affiliations:** 1 Oak, phoenix care center, Dublin, Ireland; 2 Psychiatry, poonch medical college, rawalakot, Pakistan

**Keywords:** depressive symptoms, pregnancy, husband abroad

## Abstract

**Introduction:**

Going abroad for employment is one of the common social problems which have been faced by the young males of developing countries. This included both highly qualified individuals as well as the labor class.

**Objectives:**

To determine the difference in the presence of depressive symptoms among pregnant women with husbands living abroad and those with husbands living with them in Azad Jammu and Kashmir

**Methods:**

The sample population comprised of pregnant women reporting for ante natal checkup at Amna hospital Rawalakot. Cases constituted the pregnant women with husbands living abroad while controls were the pregnant women with husbands living with them.PHQ-9 was used to record the presence and severity of depressive symptoms. Age, gestation, parity, rural or urban origin, education, level of family income, daily contact hours on telephone or what’s app, previous pregnancy loss or complications, number of years abroad and visits to home per year were associated with depressive symptoms.

**Results:**

Mean age of the study participants was 29.73±5.395 years. 66 (66%) had significant depression in the case group while 14 (14%) had in the control group (p-value<0.001). Education and rural background had significant difference among the case and control group. Less number of visits per year of husband was strongly linked with presence of depressive symptoms among the cases.

**Conclusions:**

This study showed a high frequency of depressive symptoms among pregnant women with husbands abroad as compared to those with husbands living with them. Special attention should be paid to the women whose husband had lesser number of visits to the country

